# Local ROI Reconstruction via Generalized FBP and BPF Algorithms along More Flexible Curves

**DOI:** 10.1155/IJBI/2006/14989

**Published:** 2006-02-05

**Authors:** Hengyong Yu, Yangbo Ye, Shiying Zhao, Ge Wang

**Affiliations:** ^1^ CT/Micro-CT Laboratory, Department of Radiology, University of Iowa, Iowa City, IA 52242, USA; ^2^ Department of Mathematics, University of Iowa, Iowa City, IA 52242, USA

## Abstract

We study the local region-of-interest (ROI) reconstruction problem, also referred to as the local CT problem. Our scheme includes two steps: (a) the local truncated normal-dose projections are extended to global dataset by combining a few global low-dose projections; (b) the ROI are reconstructed by either the generalized filtered backprojection (FBP) or backprojection-filtration (BPF) algorithms. The simulation results show that both the FBP and BPF algorithms can reconstruct satisfactory results with image quality in the ROI comparable to that of the corresponding global CT reconstruction.


Hindawi Publishing Corporation
International Journal of Biomedical Imaging
Volume 2006, Article ID 14989, Pages 1–7
DOI 10.1155/IJBI/2006/14989

### Local ROI Reconstruction via Generalized FBP and BPF Algorithms along More Flexible Curves

**Hengyong Yu,[1] Yangbo Ye,[1,2] Shiying Zhao,[1] and Ge Wang[1,2]**

[1] *CT/Micro-CT Laboratory, Department of Radiology, University of Iowa, Iowa City, IA 52242, USA*
[2] *Department of Mathematics, University of Iowa, Iowa City, IA 52242, USA*

Received 12 October 2005; Accepted 20 December 2005

We study the local region-of-interest (ROI) reconstruction problem, also referred to as the local CT problem. Our scheme includes two steps: (a) the local truncated normal-dose projections are extended to global dataset by combining a few global low-dose projections; (b) the ROI are reconstructed by either the generalized filtered backprojection (FBP) or backprojection-filtration (BPF) algorithms. The simulation results show that both the FBP and BPF algorithms can reconstruct satisfactory results with image quality in the ROI comparable to that of the corresponding global CT reconstruction.

Copyright © 2006 Hengyong Yu et al. This is an open access article distributed under the Creative Commons Attribution License, which permits unrestricted use, distribution, and reproduction in any medium, provided the original work is properly cited.

### 1. INTRODUCTION

It is well known that the primary disadvantage of X-ray CT is ionizing radiation which may induce cancers and cause genetic damages with a probability related to the radiation dose. Thus, reducing the dose as low as possible is a general rule for practical medical applications. One effective way is to reduce the region or volume to be imaged. For example, reconstruction from truncated projections, which is also referred to as local region reconstruction, has been extensively studied. The existent algorithms can be categorized into three categories: (1) iterative methods [1, 2] which reconstruct the local ROI by minimizing I-divergence or maximizing statistical likelihood; (2) analytic methods such as those based on wavelet-based multi-resolution analyses [3, 4]; and (3) lambda tomography methods [5–7] which recover a gradient-like function of an object function.

To reconstruct a long object such as a patient, Katsevich proposed an exact and efficient FBP reconstruction algorithm [8, 9] for a standard cone-beam helical scanning trajectory. Zou and Pan derived a BPF algorithm [10]. Later, several groups independent generalized both the FBP [11, 12] and BPF [13–18] algorithms to more general scanning loci. These results offer a new opportunity to reconstruct the local ROI from truncated data. Based on the idea of combining global and local data for improved ROI reconstruction [3, 19–21], here we propose a local region reconstruction scheme which is novel in terms of the reconstruction methods, that is, the utilization of the recently developed generalized FBP and BPF algorithms. The major characteristic is to deliver a normal radiation dose to a local ROI that may contain the cancerous tissue while applying a very low radiation dose to the structures outside the ROI.

In the following, the generalized FBP and BPF algorithms are briefly reviewed in Section 2. The ROI reconstruction scheme is described in Section 3. Simulated results and analysis are presented in Section 4. Finally, in Section 5 we conclude this paper.

### 2. REVIEW OF GENERALIZED FBP AND BPF ALGORITHMS

Based on Katsevich's FBP formula [9] for standard helical cone-beam CT, Ye and Wang derived a generalized FBP formula for exact image reconstruction from cone-beam data collected along a flexible three-dimensional (3D) curve [12]. The key step is to choose a filtering direction based on the general condition (see [12, equation 3.25]). A natural choice is the direction of the generalized PI-segment, also referred as a chord. As a result, the generalized FBP method does not require the uniqueness of the chord. In fact, it can be used to reconstruct images on any chord as long as a scanning curve runs from one endpoint of the chord to the other endpoint. We implemented this generalized formula and applied it to reconstruct images from data collected along a nonstandard saddle curve [22].

By interchanging the order of the Hilbert filtering and backprojection operation in Katsevich's FBP formula [9], Zou and Pan obtained a BPF formula for a standard helical cone-beam CT [10], which reconstructs an object only from the minimum data in the Tam-Danielsson window. Our group contributed the first proof of the general validity of the BPF formula in the case of nonstandard spirals as well as other more general curves [14, 15, 22, 23]. Independent work on exact cone-beam reconstruction in the case of a general scanning curve was also independently reported by several groups [11, 13, 17, 18]. Although there are several schemes for constructing cone-beam inversion algorithms in a general case [24–27] before these recent publications, the generalized exact cone-beam reconstruction algorithms are explicit and straightforward. Note that earlier than our SPIE paper [14], Palamodov once formulated a general inverse formula [28]. Unfortunately, his formula is not theoretically exact [29]. Since a 2D locus can be regarded as a special class of 3D curves, we can readily obtain the corresponding generalized fan-beam reconstruction formulas [30]. Therefore, in this paper, we will discuss the reconstruction of a local ROI from truncated fan-beam and cone-beam data collected along flexible 2D/3D loci.

### 3. RECONSTRUCTION SCHEME

It is well known that a reconstructed 2D ROI from local truncated projection data suffers from image cupping and intensity shifting artifacts since the local CT problem is not uniquely solvable [31]. Based on the fact that the artifacts mainly distribute in low frequencies, the local ROI reconstruction can be improved by combining the truncated local projections and very few noisy global projections. Therefore, we can deliver a normal radiation dose to the local ROI that may contain the cancerous tissue while applying a very low radiation dose to the structures outside the ROI. As shown in Figure 1, there are two types of detectors: one is a local detector (solid thick line) which collects truncated local projections at a normal radiation dose rate, and the other is a global detector (dotted thin line) which collects global projections with very low radiation dose. For practical applications, the two types of detectors can be combined into one. As illustrated in Figure 1(a), this can be physically implemented by adding some lead filters in the current pre-patient collimator. Compared to the local normal-dose projections, the global low-dose projections can be obtained in two ways: (a) reducing the number of photons for each detector aperture; (b) shortening the scanning time by reducing the number of projections. The global data and local data can be acquired in two scans [21] or the same scan with an ROI beam filtering technique [20].

If the global and local data are acquired in two scans, an interpolation procedure is required to combine the two datasets. Assume that both the local and global detectors have the same scanning geometry. The local normal-dose projections $P_{\text{local}}$ are finely sampled, while the global low-dose projections $P_{\text{global}}$ are coarsely sampled. The two datasets can be combined as

$$P^{c}_{\text{global}} = \begin{cases} P_{\text{local}} & \text{if } P_{\text{local}} \text{ is defined,} \\ P^{i}_{\text{global}} & \text{otherwise,} \end{cases} \qquad (1)$$

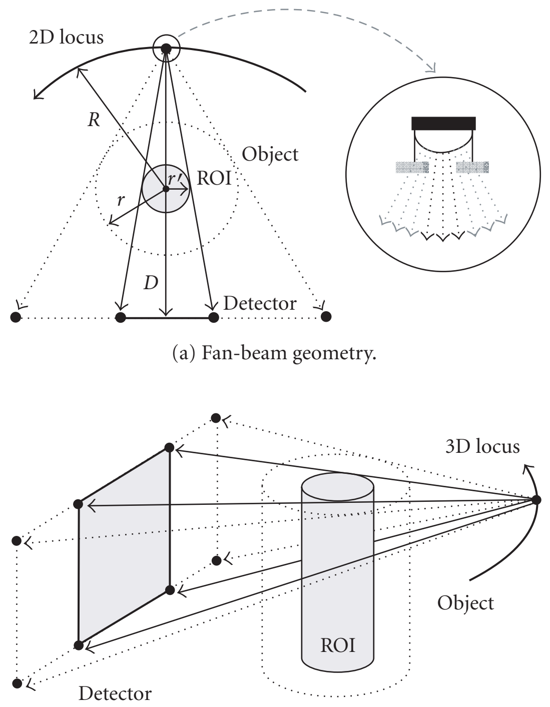

(a) Fan-beam geometry.

(b) Cone-beam geometry.

FIGURE 1: Configuration of the local region reconstruction from truncated local normal-dose data and global low-dose data. The dotted thin line represents the detector for global data while the solid thick line represents the detector for local data.

where $P^{i}_{\text{global}}$ denotes the linear interpolation of $P_{\text{global}}$ on the fine grid of $P_{\text{local}}$. The whole interpolation procedure is illustrated in Figure 2 for fan-beam geometry. Once the combined global dataset $P^{c}_{\text{global}}$ is obtained, the local ROI can be reconstructed by either the generalized FBP or BPF method. For the implementation details of the reconstruction algorithms, please refer to our previous papers [22, 23, 30]. It is pointed out that the global dataset $P^{c}_{\text{global}}$ can be directly acquired using an ROI beam filtering technique [20].

### 4. SIMULATION RESULTS

According to the analysis of our CMCT system [19], the organ dose, DOSE, is proportional to the total photons flux $\Phi$. Assume that there are $N$ photons emitted from the X-ray source to each detector aperture, the number of detectors is $S$, and the number of projections is $P$, $\Phi$ will be proportional to $N \times S \times P$, that is,

$$\text{DOSE} \propto \Phi \propto N \times S \times P. \qquad (2)$$

Hence, the ratio between the dose from global projections and that from local truncated data roughly is

$$\text{DOSE}_{\text{ratio}} = \frac{\text{DOSE}_{\text{global}}}{\text{DOSE}_{\text{local}}} = \frac{N_{\text{global}}}{N_{\text{local}}} \times \frac{S_{\text{global}}}{S_{\text{local}}} \times \frac{P_{\text{global}}}{P_{\text{local}}}, \qquad (3)$$

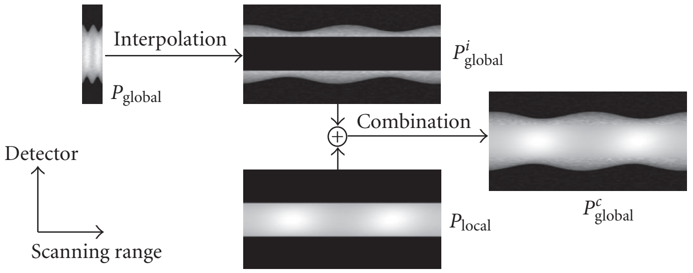

FIGURE 2: Illustration of combining local normal-dose data and global low-dose data in fan-beam geometry.

where the subscribes "global" and "local" indicate the global and local datasets, respectively.

Assume that among the $N$ photons emitted by the X-ray source only $\hat{N}$ photons arrive at the detector aperture due to attenuation in the object, and that the number of photons $\hat{N}$ obeys a Poisson distribution. Since the dose level as described by noise variance is inversely proportional to the number of the detected photons $\hat{N}$, we can simulate projections at different dose levels in terms of Poisson random variables as a function of the number of the detected photons (see the appendix). To demonstrate the performance of the proposed algorithms, we implemented an X-ray projection simulator, and the generalized FBP and BPF algorithms in Matlab on a PC with all the computational intensive part coded in C++ language.

As shown in Figure 1(a), in our first set of numerical tests, a 2D differentiable Shepp-Logan phantom (DSLP) [32] was confined in a disk of radius $r = 10$ cm, and centered at the origin of the natural coordinate system. The X-ray source was rotated along a circular locus of radius $R = 50$ cm. Collinear local and global detectors were combined with the local detector arranged in the middle of the global detector. The distance between the detector and the X-ray source was $D = 100$ cm. Along a 40.82 cm length, 500 detector elements were distributed for global data, while 250 detector elements were spanned over 20.41 cm for local data. The local detectors covered all the projections of an ROI of radius $r' = 5.08$ cm. In our simulation, 720 local truncated normal dose and 36 global low dose projections were equiangularly acquired in two scans. We set $N = 1.0 \times 10^8$ per detector element for collection of local data at the normal dose. For different low-dose levels, global data were acquired, and the local ROI reconstructed by both the FBP and BPF methods, as shown in Figure 3. Furthermore, Table 1 includes the dose ratios and signal-to-noise ratio (SNR) in the ROI.

As the baseline, the last column of Table 1 gives the case when the global dataset was obtained using the same dose level and projection number as for the local dataset. From Table 1 and Figure 3, it can be observed that (a) there were some low-frequency artifacts at the edges of the ROI reconstructed by the FBP method; (b) there were some strip artifacts along the PI-segments all over the ROI reconstructed by the BPF method; (c) the FBP method offered better image quality in terms of SNR than the BPF method; (d) both the FBP and BPF methods reconstructed satisfactory results by incorporating some low-dose global projections, at an additional cost of less than 1% purely local CT dose.

To demonstrate the flexible of the proposed scheme, our second set of numerical tests were to reconstruct a 3D local ROI from truncated cone-beam data along a nonstandard saddle curve. These tests were based on the experiments in [22]. All parameters are the same as those in [22] except the following: (a) the reconstructed object was a 3D DSLP [32]; (b) the global detector array contained $518 \times 592$ elements while the local detector array had $256 \times 592$ elements; (c) 1200 local projections and 120 global projections were acquired in two scans. The numbers of photons for local normal-dose and global low-dose scans were $N = 10^8$ and $N = 10^4$ per detector element, respectively. The dose ratio was estimated as $2.02 \times 10^{-5}$. The SNRs in the local ROI reconstructed by the FBP and BPF algorithms were 49.94 dB and 49.32 dB, respectively. Figure 4 presents representative reconstructed images.

### 5. DISCUSSION AND CONCLUSION

Since the exact FBP and BPF algorithms are utilized, the proposed scheme is different from the existent ROI beam filtering technique [20] in which the approximate Feldkamp algorithm is used. Although our interpolation method (1) appears similar to the multi-resolution analysis method (MRAM) [3, 4, 21], we emphasize that the former is different from the latter in terms of the radiation dose. Both the global and local data are typically at the same dose level (noise level) in the MRAM, while here the dose level of the global data is far lower than that of the local data. Therefore, the contribution of this paper is to combine a normal-dose local ROI scan [3, 19–21] with a low-dose global scan, and apply the exact FBP and BPF reconstruction algorithms such as those described in [12, 15].

In conclusion, we have evaluated a new local ROI reconstruction scheme from data collected along scanning curves. By combining normal-dose local projections with some low-dose global projections, we have enhanced a local normal-dose dataset to a global dataset. Both the generalized FBP and BPF algorithms have been tested to reconstruct a local ROI. The simulation results have shown that both the FBP and BPF algorithms can produce excellent results with a minimal increment to the dose needed for purely local CT.

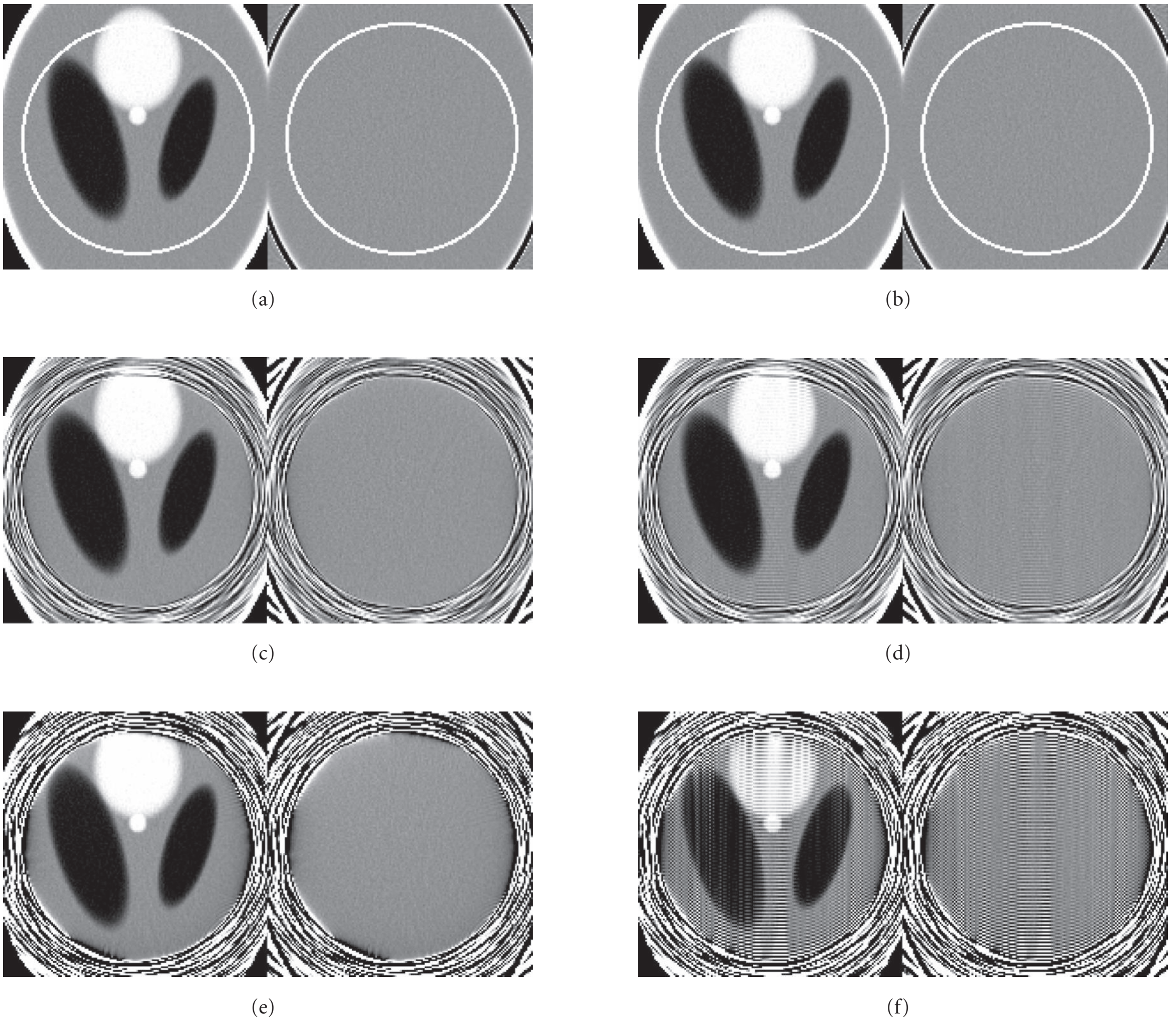

Figure 3: Reconstructed local ROI images of the DSLP with different low-dose levels for global projections. The left column was reconstructed by the FBP method while the right column by the BPF method. Each image includes a reconstructed image with a display window $[1.0, 1.04]$ and a difference image against the real image with a display window $[-0.02, 0.02]$. The global projections for the first row were collected with the same $N$ and $P$ as that for the local projections. The white circle indicates the ROI. $N$ for global data with the second and third rows were $10^5$, $10^3$, respectively.

Table 1: Dose ratios and SNRs for different low-dose levels.

| $N$ | $10^7$ | $10^6$ | $10^5$ | $10^4$ | $10^3$ | $10^2$ | |
|---|---|---|---|---|---|---|---|
| $DOSE_{ratio}$ | $10^{-2}$ | $10^{-3}$ | $10^{-4}$ | $10^{-5}$ | $10^{-6}$ | $10^{-7}$ | 2.0 |
| SNR with FBP (dB) | 61.29 | 61.12 | 60.95 | 58.06 | 49.75 | 31.00 | 61.70 |
| SNR with BPF (dB) | 61.03 | 59.78 | 55.65 | 46.89 | 37.76 | 25.86 | 61.72 |

### APPENDIX

### *Simulation of Poisson noise*

When a material is bombarded by high-speed electrons, the X-ray photons are produced. After their interactions (photoelectric, Compton, and coherent scattering) with the material, some photons are absorbed or scattered. In other words, they are attenuated when they go through the material. The attenuation process can be described by the Lambert-Beer law in the case of single energy photons. As a result, the number of the transmitted photons is a random variable obeying the Poisson distribution [33].

Assume that $N$ photons are emitted from the X-ray source towards each detector aperture, we can simulate the Poisson noise inherent with projection data from the mathematical phantom as follows.

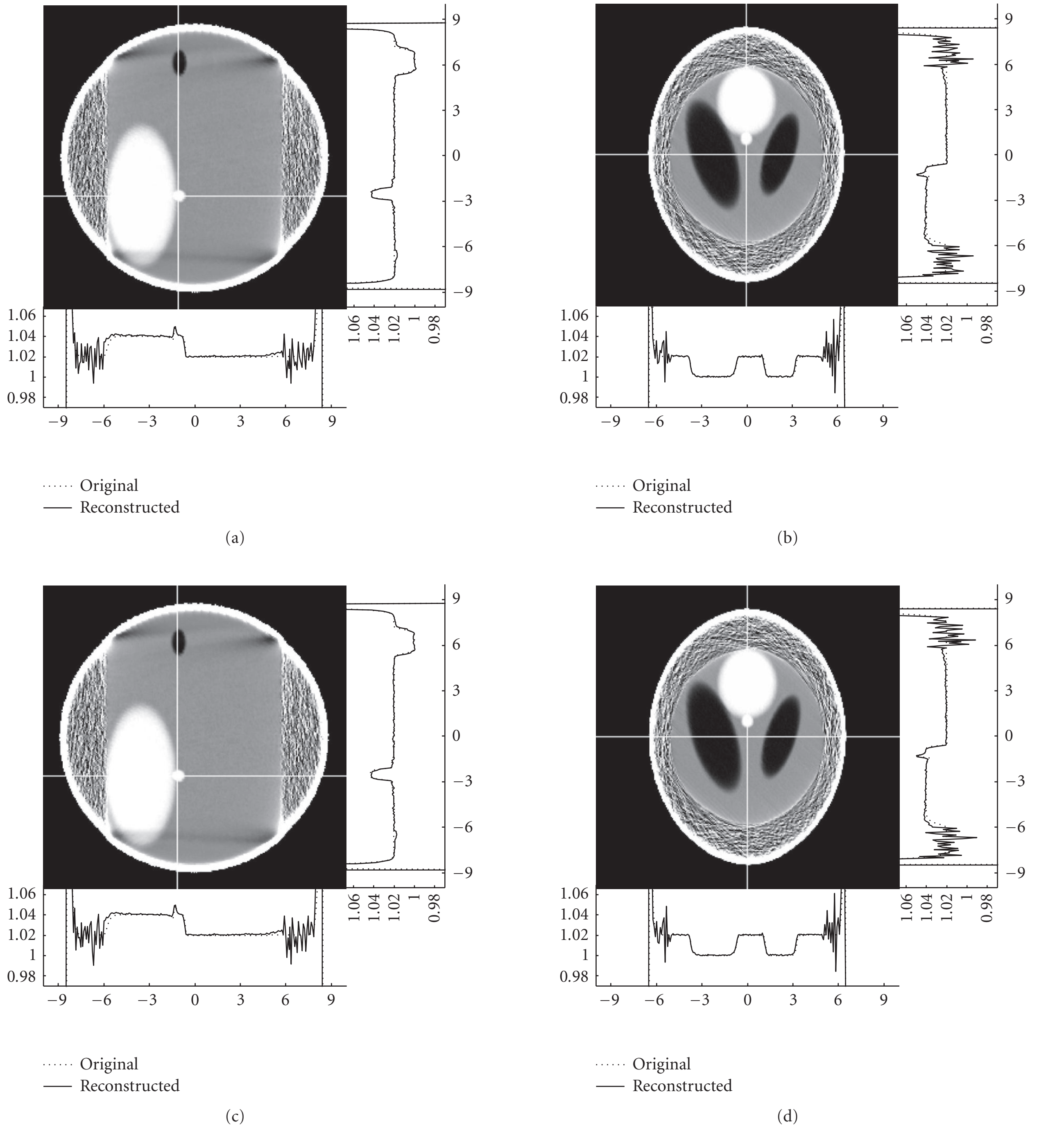

Figure 4: Typical reconstructed images of the DSLP from data collected along a nonstandard saddle curve with a display window [1.0, 1.04]. The top slices were reconstructed by the FBP method, while the bottom slices were reconstructed by the BPF method. The left and right slices are at $X = 0$ cm and $Z = -2.5$ cm, respectively. The two profiles along the white lines are plotted for each slice. The dotted and solid curves represent the original and reconstructed profiles.

*Step 1.* Compute the projection data $p$ by linear integration.

*Step 2.* Compute the expected photon number arriving at the detector, according to the Lambert-Beer law $\overline{N} = N\exp(-\mu_w p)$, where $\mu_w$ is the X-ray linear attenuation coefficient for water.

*Step 3.* Generate a Poisson random variable $\widehat{N}$, with the mean and variance being equal to $\overline{N}$.

*Step 4.* Compute noisy projection data $\widetilde{p} = (1/\mu_w)\ln(N/\widehat{N})$.

Note that X-ray projection data $p$ computed in Step 1 are based on the relative linear attenuation coefficients that have been normalized with respect to the linear attenuation coefficient for water $\mu_w$ [32]. Hence, in Step 2 the normalized projection data $p$ must be converted to have realistic magnitudes by multiplying the normalized data $p$ with the water coefficient $\mu_w$. Hence, with the formula $\overline{N} = N\exp(-\mu_w p)$, we can mimic the real X-ray attenuation process through the human head.

### ACKNOWLEDGMENT

This work is partially supported by the NIH/NIBIB Grants EB002667 and EB004287.

### REFERENCES

[1] G. Wang, D. L. Snyder, and M. W. Vannier, "Local computed tomography via iterative deblurring," *Scanning*, vol. 18, no. 8, pp. 582–588, 1996.

[2] G. Wang, M. W. Vannier, and P.-C. Cheng, "Iterative X-ray cone-beam tomography for metal artifact reduction and local region reconstruction," *Microscopy and Microanalysis*, vol. 5, no. 1, pp. 58–65, 1999.

[3] T. Olson and J. DeStefano, "Wavelet localization of the Radon transform," *IEEE Transactions on Signal Processing*, vol. 42, no. 8, pp. 2055–2067, 1994.

[4] S. Zhao and G. Wang, "Feldkamp-type cone-beam tomography in the wavelet framework," *IEEE Transactions on Medical Imaging*, vol. 19, no. 9, pp. 922–929, 2000.

[5] M. A. Anastasio, D. Shi, X. Pan, C. Pelizzari, and P. Munro, "A preliminary investigation of local tomography for megavoltage CT imaging," *Medical Physics*, vol. 30, no. 11, pp. 2969–2980, 2003.

[6] A. Faridani, E. L. Ritman, and K. T. Smith, "Local tomography," *SIAM Journal on Applied Mathematics*, vol. 52, no. 2, pp. 459–484, 1992.

[7] A. Katsevich, "Cone beam local tomography," *SIAM Journal on Applied Mathematics*, vol. 59, no. 6, pp. 2224–2246, 1999.

[8] A. Katsevich, "Theoretically exact filtered backprojection-type inversion algorithm for spiral CT," *SIAM Journal on Applied Mathematics*, vol. 62, no. 6, pp. 2012–2026, 2002.

[9] A. Katsevich, "An improved exact filtered backprojection algorithm for spiral computed tomography," *Advances in Applied Mathematics*, vol. 32, no. 4, pp. 681–697, 2004.

[10] Y. Zou and X. C. Pan, "Exact image reconstruction on PI-lines from minimum data in helical cone-beam CT," *Physics in Medicine and Biology*, vol. 49, no. 6, pp. 941–959, 2004.

[11] J. D. Pack and F. Noo, "Cone-beam reconstruction using 1D filtering along the projection of *M*-lines," *Inverse Problems*, vol. 21, no. 3, pp. 1105–1120, 2005.

[12] Y. B. Ye and G. Wang, "Filtered backprojection formula for exact image reconstruction from cone-beam data along a general scanning curve," *Medical Physics*, vol. 32, no. 1, pp. 42–48, 2005.

[13] J. D. Pack, F. Noo, and R. Clackdoyle, "Cone-beam reconstruction using the backprojection of locally filtered projections," *IEEE Transactions on Medical Imaging*, vol. 24, no. 1, pp. 70–85, 2005.

[14] Y. B. Ye, S. Zhao, H. Y. Yu, and G. Wang, "Exact reconstruction for cone-beam scanning along nonstandard spirals and other curves," in *Developments in X-Ray Tomography IV*, vol. 5535 of *Proceedings of SPIE*, pp. 293–300, Denver, Colo, USA, August 2004.

[15] Y. B. Ye, S. Zhao, H. Y. Yu, and G. Wang, "A general exact reconstruction for cone-beam CT via backprojection-filtration," *IEEE Transactions on Medical Imaging*, vol. 24, no. 9, pp. 1190–1198, 2005.

[16] S. Zhao, H. Y. Yu, and G. Wang, "A unified framework for exact cone-beam reconstruction formulas," *Medical Physics*, vol. 32, no. 6, pp. 1712–1721, 2005.

[17] T. L. Zhuang, S. Leng, B. E. Nett, and G.-H. Chen, "Fan-beam and cone-beam image reconstruction via filtering the backprojection image of differentiated projection data," *Physics in Medicine and Biology*, vol. 49, no. 24, pp. 5489–5503, 2004.

[18] Y. Zou and X. C. Pan, "An extended data function and its generalized backprojection for image reconstruction in helical cone-beam CT," *Physics in Medicine and Biology*, vol. 49, no. 22, pp. N383–N387, 2004.

[19] G. Wang, S. Zhao, H. Y. Yu, et al., "Design, analysis and simulation for development of the first clinical micro-CT scanner," *Academic Radiology*, vol. 12, no. 4, pp. 511–525, 2005.

[20] R. N. Chityala, K. R. Hoffmann, D. R. Bednarek, and S. Rudin, "Region of interest (ROI) computed tomography," in *Medical Imaging 2004: Physics of Medical Imaging*, vol. 5368 of *Proceedings of SPIE*, pp. 534–541, San Diego, Calif, USA, February 2004.

[21] S. Azevedo, P. Rizo, and P. Grangeat, "Region-of-interest cone-beam computed tomography," in *Proceedings of the International Meeting on Fully Three-Dimensional Image Reconstruction in Radiology and Nuclear Medicine (3D '95)*, pp. 381–385, Aix les Bains, France, July 1995.

[22] H. Y. Yu, S. Zhao, Y. B. Ye, and G. Wang, "Exact BPF and FBP algorithms for nonstandard saddle curves," *Medical Physics*, vol. 32, no. 11, pp. 3305–3312, 2005.

[23] H. Y. Yu, Y. B. Ye, S. Zhao, and G. Wang, "A backprojection-filtration algorithm for nonstandard spiral cone-beam CT with an *n*-PI-window," *Physics in Medicine and Biology*, vol. 50, no. 9, pp. 2099–2111, 2005.

[24] P. Grangeat, "Mathematical framework of cone beam 3D reconstruction via the first derivative of the Radon transform," in *Mathematical Methods in Tomography*, G. T. Herman, A. K. Louis, and F. Natterer, Eds., pp. 66–97, Springer, New York, NY, USA, 1991.

[25] A. Katsevich, "A general scheme for constructing inversion algorithms for cone beam CT," *International Journal of Mathematics and Mathematical Sciences*, vol. 2003, no. 21, pp. 1305–1321, 2003.

[26] B. D. Smith, "Image reconstruction from cone-beam projections: necessary and sufficient conditions and reconstruction methods," *IEEE Transactions on Medical Imaging*, vol. 4, no. 1, pp. 14–25, 1985.

[27] H. K. Tuy, "An inversion formula for cone-beam reconstruction," *SIAM Journal on Applied Mathematics*, vol. 43, no. 3, pp. 546–552, 1983.

[28] V. P. Palamodov, "Reconstruction from ray integrals with sources on a curve," *Inverse Problems*, vol. 20, no. 1, pp. 239–242, 2004.

[29] H. Y. Yu, Y. B. Ye, S. Y. Zhao, and G. Wang, "Studies on Palamodov's algorithm for cone-beam CT along a general curve," to appear in Inverse Problems.

[30] H. Y. Yu, et al., "Fan-beam reconstruction with compensation for in-plane motion based on data consistency," *IEEE Transactions on Medical Imaging*, under review.

[31] F. Natterer, *The Mathematics of Computerized Tomography*, John Wiley & Sons, New York, NY, USA, 1986.

[32] H. Y. Yu, S. Zhao, and G. Wang, "A differentiable Shepp-Logan phantom and its applications in exact cone-beam CT," *Physics in Medicine and Biology*, vol. 50, no. 23, pp. 5583–5595, 2005.

[33] A. C. Kak and M. Slaney, *Principles of Computerized Tomographic Imaging*, SIAM, Philadelphia, Pa, USA, 2nd edition, 2001.

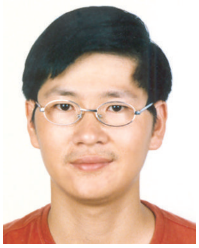

**Hengyong Yu** received his B.S. degree in information science and technology and his Ph.D. degree in information and communication engineering from Xi'an Jiaotong University, Xi'an, Shaanxi, China, in July 1998 and June 2003, respectively. He was an Instructor and Associate Professor with the College of Communication Engineering, Hangzhou Dianzi University, Hangzhou, Zhejiang, China, from July 2003 to September 2004. Currently, he is a Research Fellow with the CT/Micro-CT Laboratory, Department of Radiology, University of Iowa, Iowa City, USA. His interests include computed tomography and medical image processing. He has authored or coauthored more than 30 peer-reviewed papers. He serves as a Guest Editor of the International Journal of Biomedical Imaging for the special issue entitled *Development of Computed Tomography Algorithms*. He is a Member of the IEEE and the Chinese Institute of Electronics. He was honored for an outstanding doctorial dissertation by Xi'an Jiaotong University, and received the first prize for a best natural science paper from the Association of Science and Technology of Zhejiang Province.

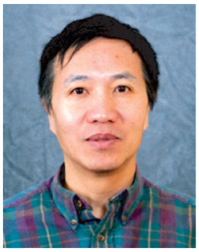

**Yangbo Ye** is a Professor of Mathematics and Professor of Radiology at the University of Iowa. In medical imaging, his research is focused on reconstruction formulas and algorithms. His recent research work includes proofs of exact reconstruction of cone-beam data scanned along an arbitrary locus in the filtered backprojection and backprojection filtration framework.

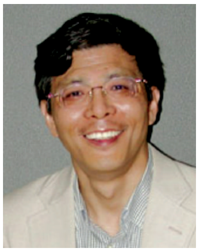

**Shiying Zhao** received his B.A. degree in mechanical engineering from University of Science and Technology of China in 1982, M.S. degree in mechanical engineering from Shanghai Jiao Tong University, China, in 1985, and Ph.D. degree in mathematics from University of South Carolina, Columbia, South Carolina, in 1991. Currently, he is an Associate Professor with Department of Mathematics and Computer Science, University of Missouri - St. Louis. His research interests include X-ray tomography, wavelet analysis, and imaging processing.

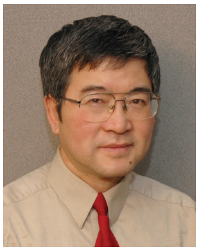

**Ge Wang** received his B.E. degree in electrical engineering from Xidian University, Xi'an, China, in 1982, his M.S. degree in remote sensing from the Graduate School of Academia Sinica, Beijing, China, in 1985, and his M.S. and Ph.D. degrees in electrical and computer engineering from the State University of New York, Buffalo, in 1991 and 1992. He was the Instructor and Assistant Professor with the Department of Electrical Engineering, Graduate School of Academia Sinica in 1984–1988, the Instructor and Assistant Professor with Mallinckrodt Institute of Radiology, Washington University, St. Louis, Missouri, in 1992–1996. He was an Associate Professor with the University of Iowa from 1997 to 2002. Currently, he is a Professor with the Departments of Radiology, Biomedical Engineering, Civil Engineering, and Mathematics, and the Director of the Center for X-ray and Optical Tomography, University of Iowa. His interests include computed tomography, bioluminescence tomography, and systems biomedicine. He has authored or coauthored over 300 journal articles and conference papers, including the first paper on spiral/helical cone-beam CT and the first paper on bioluminescence tomography. He is the Editor-in-Chief for the International Journal of Biomedical Imaging, and the Associate Editor for the IEEE Transactions on Medical Imaging and Medical Physics. He is an IEEE Fellow and an AIMBE Fellow. He is also recognized by a number of awards for academic achievements.

